# Operative Versorgungsstrategien bei interprothetischen Femurfrakturen

**DOI:** 10.1007/s00132-025-04686-9

**Published:** 2025-07-29

**Authors:** Christian Ries, Holger Bäthis, Stephan Kirschner, Patrick Gerhardt, Eva Goedecke, Tim Rolvien, Frank Timo Beil

**Affiliations:** 1 Klinik für Unfallchirurgie und Orthopädie, Lehrstuhl für Orthopädie, Universitätsklinikum Hamburg-Eppendorf, Martinistraße 52, 20246 Hamburg, Deutschland; 2 Klinik für Orthopädie, Unfallchirurgie und Sporttraumatologie, Klinikum Köln-Merheim, Lehrstuhl der Universität Witten-Herdecke, Köln, Deutschland; 3 Klinik für Orthopädie, ViDia-Kliniken, Karlsruhe, Deutschland; 4 Klinik für Orthopädie und Unfallchirurgie, Agaplesion Markus Krankenhaus, Frankfurt am Main, Deutschland; 5 Helios ENDO-Klinik Hamburg, Hamburg, Deutschland; 6 AE-Komitee „Frakturendoprothetik und periprothetische Frakturen“, Deutsche Gesellschaft für Endoprothetik (AE), Freiburg, Deutschland

**Keywords:** Osteoporose, Osteosynthese, Periprothetische Fraktur, Revisionschirurgie, Hüfttotalendoprothese, Osteoporosis, Osteosynthesis, Periprosthetic fracture, Revision surgery, Total hip arthroplasty

## Abstract

Durch den demografischen Wandel ist eine weitere Zunahme von endoprothetischen Versorgungen anzunehmen. Entsprechend ist, wie bereits in den letzten Jahren zu beobachten, mit einem weiteren Anstieg periprothetischer Frakturen zu rechnen. Die interprothetische Femurfraktur ist eine schwerwiegende Komplikation bei ipsilateral einliegender Hüft- und Kniegelenkendoprothese. Wenngleich die Inzidenz der interprothetischen Fraktur gering ist, so stellt das Frakturmanagement an den Behandelnden mitunter hohe Ansprüche. In den Entscheidungsprozess bei der Frakturversorgung müssen neben der verbliebenen Implantatstabilität auch die Knochenqualität und etwaige Komorbiditäten mit einbezogen werden. Die Expertise der Behandelnden in verschiedenen Osteosyntheseverfahren sowie in der Revisionsendoprothetik scheint unabdingbar. Unter Berücksichtigung verschiedener patientenspezifischer Faktoren gibt der folgende Beitrag einen orientierenden Überblick zur operativen Versorgung interprothetischer Frakturen.

## Abstract (en)

Due to demographic changes, a further increase in the number of arthroplasty procedures can be anticipated. As has been observed in recent years, a further increase in periprosthetic fractures is to be expected. An interprosthetic femoral fracture is a serious complication in cases of ipsilateral hip and knee endoprostheses. Although the incidence of interprosthetic fractures is low, fracture management can be highly demanding. In addition to the remaining implant stability, bone quality and comorbidities must also be taken into account in the decision-making process of fracture treatment. The surgeon’s expertise in various osteosynthesis procedures and revision arthroplasty techniques appears to be indispensable. Taking into account various patient-specific factors, the following article provides an overview of the surgical treatment options for interprosthetic fractures.

Die interprothetische Femurfraktur ist eine schwerwiegende Komplikation nach endoprothetischem Gelenkersatz von Hüfte und Knie. Wenngleich die Inzidenz gering ist, so stellt das Frakturmanagement an den Behandelnden mitunter hohe Ansprüche. Die Expertise in verschiedenen osteosynthetischen Verfahren sowie in der komplexen Revisionsendoprothetik erscheint unabdingbar. Diese Übersichtsarbeit beschreibt Strategien der interprothetischen Frakturversorgung.

## Einleitung

Auch im Jahr 2023 wurde in Deutschland ein weiterer Zuwachs an endoprothetischen Versorgungen verzeichnet. Die Anzahl der gemeldeten Kniegelenkendoprothesen im EPRD betrug mehr als 170.000, die der Hüftgelenktotalendoprothesen sogar über 200.000 [1]. Das durchschnittliche Patientenalter bei Primärimplantation von Hüft- und Kniegelenkendoprothesen liegt in Deutschland bei 71 Jahren bzw. 69 Jahren [1]. Der erfolgreich implantierte Gelenkersatz ermöglicht den Patienten durch die verbesserte Schmerzsymptomatik, Funktionalität und Mobilität eine aktive Teilnahme am gesellschaftlichen Alltag. Unterdessen zeigt die Analyse der allgemeinen Sterbetafel des statistischen Bundesamtes einen kontinuierlichen Anstieg der Lebenserwartung in Deutschland, wenngleich durch die Corona-Pandemie nicht mehr so deutlich wie in den vorvergangenen Jahrzehnten [2]. Somit ist mittel- bis langfristig ebenso mit einer Zunahme implantatassoziierter Komplikationen zu rechnen. Während die periprothetische Femurfraktur (PFF) des Hüftgelenkes mittlerweile mit 15,8 % der dritthäufigste Grund für einen Revisionseingriff ist (Lockerung 22,7 %; Infektion 16,4 %), so wird der frakturbedingte Folgeeingriff am endoprothetisch ersetzten Kniegelenk mit nur 3,8 % beziffert [1]. Im Bereich des Kniegelenkes handelt es sich überwiegend (80 %) um distale PFF [3]. Für die PFF des Hüftgelenks (Typ Vancouver B und C) zeigt sich ein Altersgipfel zwischen 80 und 89 Jahren [4]. Bekannte Risikofaktoren für eine PFF sind neben dem Alter, weiblichem Geschlecht, Osteoporose ebenso inflammatorische Arthritiden und lokale, implantatassoziierte Osteolysen und Knochendefekte [5, 6]. Bei vorbestehender knöcherner Veränderung im Prothesenbereich, wie beispielsweise einer Lockerung der Komponenten, wäre auch ein inadäquates Trauma im Sinne einer pathologischen Fraktur denkbar. Dem Behandelnden sollte daher die erweiterte, prothesenspezifische Anamnese (z. B. „unhappy hip“ [7]) als Hilfestellung dienen und ist stets zu erheben.

Bei periprothetischen Femurfrakturen handelt es sich in 5–7 % der Fälle um eine interprothetische Femurfraktur (IFF) [8–11]. Die IFF kann als Subgruppe der PFF gesehen werden und liegt suprakondylär im Femurschaftbereich, dort wo üblicherweise die PFF Vancouver-Typ C bzw. bei einliegendem Kniegelenkersatz Frakturen vom Typ Rorabeck II lokalisiert sind [12, 13]. Beide Frakturentitäten (Vancouver-Typ C und Rorabeck-Typ II) beschreiben einen festen Sitz der einliegenden Implantate. Je nach Frakturmorphologie ist jedoch bei IFF hiervon abweichend auch eine Lockerung des Implantates möglich, welches es im Vorfeld dezidiert zu prüfen gilt [10, 11]. Im Fall der IFF müssen in den Entscheidungsprozess bei der Frakturversorgung neben der verbliebenen Implantatstabilität auch die Knochenqualität und etwaige Komorbiditäten mit einbezogen werden. Die Expertise der Behandelnden in verschiedenen Osteosyntheseverfahren sowie in der Revisionsendoprothetik scheint unabdingbar. Der folgende Beitrag gibt einen orientierenden Überblick mit spezifischem Fokus auf die IFF.

## Was sind potenzielle Risiken für eine IFF?

Es stellt sich die Frage, welche Faktoren das Risiko für eine IFF erhöhen. Liegt beispielsweise eine Hüfttotalendoprothese ein und es steht die endoprothetische Versorgung des Kniegelenkes an, so kann mitunter bei einer femoral schaftverankerten Kniegelenkprothese der Abstand zwischen den beiden intramedullär einliegenden Prothesenschäften relevant werden. Lipof et al. [14] analysierten insgesamt 23 IFF (Ø 82 Jahre, 78 % Frauen, 26 % zementierte Implantate, 30 % Osteoporose). Als radiologische Risikofaktoren – im Vergleich zu einem nicht frakturierten Kontrollkollektiv – konnten von Lipof et al. ein breiter femoraler Markraum sowie eine ausgedünnte Kortikalis auf Höhe des femoralen Isthmus ausgemacht werden. Diese Beobachtung deckt sich mit den Studienergebnissen von Valle Cruz et al. [15], welche in ihrer Arbeit röntgenologisch unter anderem den Markraumdurchmesser sowie die kortikale Dicke unmittelbar unterhalb der Prothesenspitze bzw. dem Knochenzement vermessen haben. Die Autoren schlussfolgerten anhand ihrer Daten (68 Patienten, 82 % Frauen), dass ein größerer femoraler Markraum distal der Schaftspitze das Risiko für eine Fraktur erhöht.

Iesaka et al. [16] analysierten an Sawbone-Femora mit „press fit“ implantierten Schäften mit halbrunden Schaftspitzen den interprothetischen Abstand (1/10/23/85 mm) im Rahmen einer Finite-Elemente-Studie. Selbst bei einem interprothetischen Abstand von 5 mm schien dieser auf den femoralen Stress keinen Einfluss zu haben. Eine Reduktion der kortikalen Dicke sowie gelockerte Prothesenschäfte erhöhten allerdings in der Simulation den interprothetischen „Biegestress“ bzw. zeigten einen „Stress-Peak“ im Bereich der Schaftspitze. In einer Studie von Weiser et al. [17] wurden 18 humane „fresh frozen“ Femora (Ø 62,4 Jahre) anhand von gematchten Gruppen (gematcht bzgl. BMD und kortikaler Dicke/Fläche) und definierten interprothetischen Abständen (35/80/160 mm) im Hinblick auf die knöcherne Versagenskraft mittels Vier-Punkt-Biegebelastung analysiert. In der biomechanischen Testung konnte kein signifikanter Unterschied hinsichtlich der aufzubringenden Kraft bei unterschiedlichen interprothetischen Abständen festgestellt werden. Hingegen zeigte sich eine signifikante Korrelation zwischen der kortikalen Dicke und der „load to failure“. Die Autoren schlussfolgerten, dass der interprothetische Abstand weniger relevant zu sein scheint als vielmehr die kortikale Dicke. Somit unterstützt auch die biomechanische Arbeit von Weiser et al. [17] die rein radiologischen Vermessungen von Lipof et al. [14] und Valle Cruz et al. [15].

Nichtsdestotrotz verändern sich die Eigenschaften des proximalen Femurs nach Schaftimplantation hinsichtlich der knöchernen Versagenskraft mit einer Reduktion dieser um bis zu 32 % [18], was das Frakturrisiko konsekutiv erhöht. Neben dem Risiko einer interprothetischen Fraktur bei ipsilateral einliegender Hüft- und Kniegelenkendoprothese analysierte die Arbeitsgruppe um Lehmann et al. [19] ergänzend das Risiko für unterschiedliche, im Femur einliegende Implantate. Hierfür wurde anhand von 30 humanen „fresh frozen“ Femora (Ø 76,3 Jahre) die knöcherne Versagenskraft bei einliegender Hüfttotalendoprothese mit einem retrograden Marknagel, einer femoral schaftverankerten Kniegelenkprothese sowie einer unterhalb und einer oberhalb der femoralen Komponente endenden Plattenosteosynthese verglichen. In der biomechanischen Testung zeigte sich das größte Risiko einer interimplantären Fraktur bei einliegender Hüfttotalendoprothese und retrogradem Marknagel. Bei sowohl fest verankerter Hüfttotalendoprothese als auch femoral schaftverankerter Knieprothese zeigte sich hingegen kein erhöhtes Frakturrisiko.

Wenngleich die kortikale Dicke und die Weite des femoralen Markraumes einen wichtigen Einfluss auf das interprothetische Frakturrisiko zu haben scheinen, so ist für den Behandelnden die Frage nach einer potenziellen „safe zone“ zwischen zwei intramedullär verankerten Prothesen ebenso interessant. Eine biomechanische Arbeit von Soenen et al. [20] analysierte in einer Finite-Elemente-Studie anhand von auf CT-Daten basierten 3‑D-gedruckten Femora den Abstand zwischen den Schaftspitzen bei einliegender Hüfttotalendoprothese und femoral schaftverankerter Kniegelenkprothese. Die Hüfttotalendoprothese (Polarstem System, Smith&Nephew, Memphis, TN, USA) wurde „press fit“, zementfrei implantiert. Die femorale Komponente des Kniegelenkes wurde zementiert, der Prothesenschaft nicht (Legion Revision Knee System, Smith&Nephew). In der biomechanischen Testung wurde die Belastung bei „normalem Gang“, einem seitlichen Sturz sowie bei einer Vier-Punkt-Biegebelastung getestet. Die kortikale Dicke bzw. der femorale Markraum wurden nicht vermessen.

Der interprothetische Abstand (von 5–15 cm) zeigte sich hinsichtlich des Frakturrisikos sowohl für den im simulierten Modell „normalen“ Knochen als auch für den „osteoporotischen“ Knochen unter einer axialen Belastung („normaler Gang“) nicht entscheidend. Ebenso konnte bei den „seitlichen Stürzen“ kein Unterschied des Frakturrisikos zwischen den verschiedenen interprothetischen Abständen detektiert werden. Allerdings zeigte sich in den Analysen, dass der osteoporotische Knochen im Vergleich zum im Modell „physiologisch gesunden“ Knochen frakturanfälliger ist. Hingegen konnte im Rahmen der Vier-Punkt-Biegebelastung zwischen einem interprothetischen Abstand von 9 und 11 cm sowohl für den „physiologisch gesunden“, als auch für den „osteoporotischen“ Knochen eine Verdopplung des interprothetischen Frakturrisikos aufgezeigt werden. Aus diesen Ergebnissen kann geschlussfolgert werden, dass ein interprothetischer Abstand über 11 cm das Frakturrisiko reduziert und wenn möglich angestrebt werden sollte. Wird dieser Abstand unterschritten, kann unter Risiko-Nutzen-Abwägung eine ergänzende Plattenosteosynthese erwogen werden, um das individuelle interprothetische Frakturrisiko zu reduzieren.

## Wie lassen sich IFF klassifizieren und welche Handlungsstrategien lassen sich ableiten?

Die Vancouver-Klassifikation beschreibt die proximalen PPF unter Berücksichtigung der Frakturlokalisation sowie der verbliebenen Fixierung der femoralen Komponente. Typ-C-Frakturen sind unterhalb des festsitzenden Schaftes lokalisiert und beschreiben am ehesten die Frakturlage bei einer IFF. Bei der periprothetischen Fraktur rund um das Kniegelenk hat sich die Klassifikation nach Rorabeck etabliert [21]. Hier wäre die suprakondylär gelegene Fraktur als Typ II definiert, wohlgemerkt mit fest einliegender femoraler Komponente. Während die Frakturtypen Vancouver A bis C analog zum neueren Unified Classification System (UCS) [22] sind, welches darauf abzielt eine einheitliche Klassifikation für alle Arten von periprothetischen Frakturen zu ermöglichen, so ist die IFF in der UCS als Typ D definiert. Eine weiterführende Einschätzung der Frakturmorphologie bzw. der Prothesenstabilität erfolgt bei dieser Klassifikation der IFF allerdings nicht.

Pires et al. [23] publizierten 2014 eine eigene Klassifikation von IFF und unterschieden, je nach einliegendem Implantat und Frakturlokalisation, drei Hauptgruppen. Typ I beschreibt die IFF angrenzend an die Hüfttotalendoprothese und Typ II im Bereich eines einliegenden bikondylären Oberflächenersatzes. Typ III hingegen klassifiziert die IFF bei einliegender Hüfttotalendoprothese und femoral schaftverankerter Kniegelenkprothese. Bei allen drei Typen werden Subtypen A bis D unterschieden. Während bei Typ I und II vorwiegend die Festigkeit der einliegenden Komponente durch die Subtypen beschrieben wird, so wird bei Typ III neben der Festigkeit auch die Knochenvitalität und das interprothetische Frakturmuster berücksichtigt. Eine orientierende Übersicht gibt Tab. 1. Unter Berücksichtigung der vorgenommen Fraktureinteilung leiteten Pires et al. [23] Behandlungsstrategien für die jeweilige Form der IFF ab. Die Arbeitsgruppe konstatierte, dass für die operative Versorgung von IFF unter anderem Einflussfaktoren wie hohes Patientenalter, reduzierte Knochenqualität, Komorbiditäten, kleine interprothetische Fragmente, Knochenverluste und auch gelockerte Implantate entscheidende Faktoren sind.

Pires et al. [24] validierten ihren 2014 publizierten Algorithmus und erachteten ihre Versorgungsstrategie als klinisch sinnvollen Leitfaden. Allerdings wird die vergleichsweise geringe Inter- und Intraobserver-Reliabilität der Klassifikation von anderen Autoren kritisch bemängelt, wodurch die Empfehlung ausgesprochen wurde, diese in der Klinik sowie Wissenschaft eher mit Zurückhaltung anzuwenden [25, 26]. Ein Kritikpunkt ist, dass die Detektion einer Prothesenlockerung alleinig am Röntgenbild und auch mit einer erweiterten Schnittbildgebung nicht immer abschließend möglich ist, was die korrekte präoperative Frakturklassifikation erschwert. Es bleibt unstrittig, dass die Versorgung von IFF anspruchsvoll ist. Ungeachtet dessen erscheint die Expertise der Behandelnden in verschiedenen osteosynthetischen Verfahren sowie in der komplexen Revisionsendoprothetik unabdingbar. Die Versorgung von IFF ist nicht zuletzt aufgrund ihrer geringen Inzidenz und der somit anzunehmenden geringen Anzahl von unterjährigen Versorgungen eines jeden Krankenhauses sowie dem damit einhergehenden Mangel an klinischen Daten und wissenschaftlicher Literatur herausfordernd [27, 28]. Die Arbeitsgruppe um Mamczak [28] publizierte beispielsweise im Jahr 2010 eine Arbeit mit insgesamt 26 IFF, welche in zwei Zentren über einen Zeitraum von 20 Jahren versorgt wurden. Auf jedes Zentrum fiel somit weniger als eine Versorgung einer IFF pro Jahr. Bonnevialle et al. [29] schätzten in ihrer aktuellen Studie aus dem Jahr 2019 die Inzidenz der IFF auf etwa 1–5 Fälle pro Jahr pro Traumazentrum, welches mit anderen aktuellen Arbeiten in Einklang steht [8].

**Tab. 1:** Klassifikation der interprothetischen Femurfraktur in Anlehnung an Pires et al. [23]

Typ		Subtyp	
*I*	Interprothetisch angrenzend an die Hüfttotalendoprothese	*A*	Beidseits nicht-gelockerte Implantate
		*B*	Gelockerte Hüftkomponente, nicht-gelockerter Oberflächenersatz
		*C*	Nicht-gelockerte Hüftkomponente, gelockerter Oberflächenersatz
		*D*	Beidseits gelockerte Implantate
*II*	Interprothetisch angrenzend an die Kniegelenkprothese (bikondylär)	*A*	Beidseits nicht-gelockerte Implantate
		*B*	Gelockerte Hüftkomponente, nicht-gelockerter Oberflächenersatz
		*C*	Nicht-gelockerte Hüftkomponente, gelockerter Oberflächenersatz
		*D*	Beidseits gelockerte Implantate
*III*	Interprothetisch bei femoral schaftverankerter Kniegelenkprothese	*A*	Beidseits nicht-gelockerte Implantate mit gutem interprothetischem Knochen
		*B*	Beidseits nicht-gelockerte Implantate mit interprothetischem Knochenverlust
		*C*	Gelockerte Prothese (Hüfte, Knie oder beide) mit gutem interprothetischem Knochen
		*D*	Gelockerte Prothese (Hüfte, Knie oder beide) mit interprothetischem Knochenverlust

Wenngleich derzeit keine universell anwendbaren Klassifikationen der IFF und entsprechende Handlungsalgorithmen existieren, so kann der Algorithmus von Pires et al. [23] doch als orientierender Ansatz und Versuch einer vereinheitlichenden Klassifikation verstanden werden. Es gibt letztendlich zu viele verschiedene Frakturmorphologien und individuelle Patientencharakteristika, als dass sich dies vereinfacht in einer allgemeingültigen Handlungsempfehlung abbilden ließe. Vielmehr bedarf es einer individuellen operativen Behandlungsstrategie [30]. Im Folgenden werden verschiedene Frakturentitäten in Anlehnung an die Klassifikation von Pires et al. [23] exemplarisch diskutiert. Das operative Ziel ist die Rekonstruktion der Femurlänge und des femoralen Alignments, wobei gleichzeitig die Funktion der angrenzenden Prothesen erhalten und eine feste Prothesenverankerung sichergestellt werden soll, sodass eine rasche Mobilisation des Patienten erfolgen kann.

Grundsätzlich gilt es, bei Prothesenrevisionen einen periprothetischen Infekt auszuschließen. Die akute Fraktursituation erlaubt jedoch nicht, die Langzeitbebrütung nach erfolgter Punktion abzuwarten. Die operative Versorgung darf sich durch eine etwaige Infektdiagnostik nicht verzögern. Dennoch kann die präoperative Punktion erwogen werden. Wenngleich die Zellzahl durch die Fraktursituation und die Einblutung gegebenenfalls falsch hoch ist, so stellen die Zelldifferenzierung und der Leukozytenesterasetest ergänzend eine rasche Diagnostik mit akzeptabler Sensitivität dar [31]. Ungeachtet dessen sollten intraoperativ Proben zur histopathologischen und mikrobiologischen Analyse entnommen werden. Unter Berücksichtigung des perioperativen Risikos bei komplexen prothetischen Wechseloperationen in der Akutsituation kann postoperativ eine empirische Antibiose erwogen werden. Die Antibiose kann bis zum Erhalt der mikrobiologischen Ergebnisse fortgeführt werden und ist dann bei fehlendem Nachweis pathogener Keime abzusetzen.

**Abb. 1:**
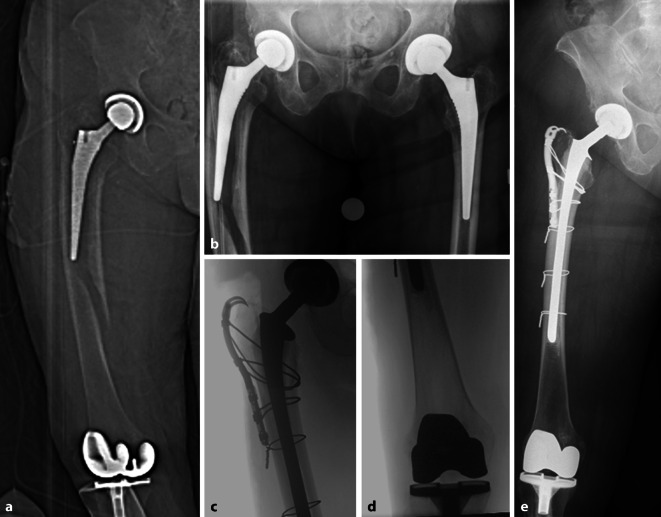
Fallbeispiel: 86 Jahre, weiblich. **a,** **b** Sturzereignis mit periprothetischer Femurschaftfraktur und gelockerter femoraler Schaftkomponente (Vancouver-Typ B2) sowie nach distal interprothetisch auslaufender Fraktur (Unified-Classification-System-Typ D). Fest einliegender bikondylärer Oberflächenersatz des Kniegelenkes. **c,** **d** Intraoperative Kontrolle nach einzeitiger Schaftrevision unter Verwendung eines zementierten Langschaftes (Fa. Link, Typ SPII (Fa. Link, Norderstedt, Deutschland)) und ORIF („open reduction and internal fixation“) des Femurschaftes mittels drei freier 1,5 mm Drahtcerclagen. Bei zudem vorliegender Fraktur des Trochanter major erfolgte additiv die Osteosynthese mit einer Krallenplatte (Fa. Smith&Nephew). **e** Postoperative Röntgenkontrolle. Freigabe der schmerzadaptierten Belastung unter physiotherapeutischer Anleitung

Bei vorliegender IFF mit gelockerter Hüfttotalendoprothese und festsitzendem bikondylärem Oberflächenersatz des Kniegelenkes handelt es sich nach Pires et al. [23] um einen *Typ IB*. In diesem Fall ist die Revision des Prothesenschaftes der einliegenden Hüfttotalendoprothese indiziert. Hinsichtlich der operativen Versorgungsstrategie bei periprothetischen Femurfrakturen mit gelockertem Prothesenschaft sind zementfreie und zementierte Revisionen zu diskutieren. Beide Verankerungsarten werden in der Literatur häufig verwendet, ohne dass sich signifikante Unterschiede ableiten lassen. Der Vorteil von zementfreien Schäften in modularer Ausfertigung wird von der Literatur aktuell nicht gestützt [32]. Ein wichtiges Ziel sollte insbesondere beim geriatrischen Patienten zur Vermeidung von internistischen Komplikationen die postoperative Vollbelastung sein. Je nach Frakturmorphologie kann die additive Verwendung von Plattenosteosynthesen und/oder Cerclagen indiziert sein (Abb. 1).

**Abb. 2:**
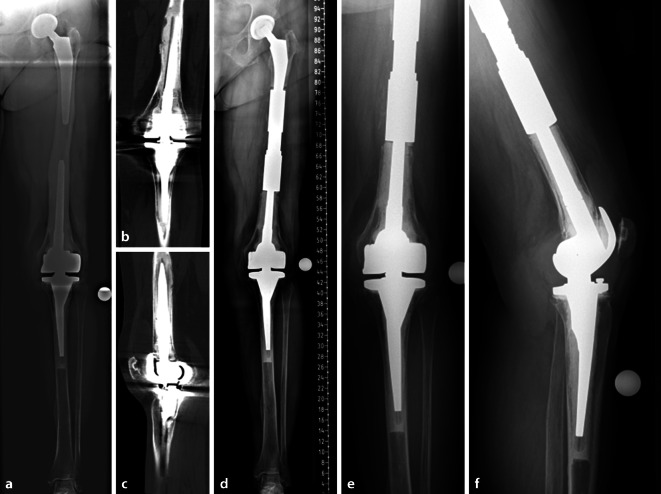
Fallbeispiel: 81 Jahre, weiblich. **a** Röntgen Ganzbeinstandaufnahme. Festsitzende teilzementierte Hüfttotalendoprothese sowie einliegende femoral und tibial schaftverankerte, zementierte gekoppelte Kniegelenkendoprothese. Der femorale Abstand zwischen den Prothesenspitzen beträgt weniger als 11 cm. Der Isthmus des Markraumes ist eher weit und die kortikale Begrenzung tendenziell ausgedünnt. Um die femorale Schaftspitze der einliegenden Knieprothese zeigt sich ein kortikales „Bowing“ im Sinne einer Stressreaktion. **b,** **c** Drei Jahre nach Implantation zeigt sich – in Korrelation zur klinischen Symptomatik – in der CT am ehesten eine interprothetische Insuffizienzfraktur. **d–f** Postoperative Röntgenkontrolle nach Revisionsoperation mittels Rescue-Sleeve (Fa. Link) unter Erhalt der einliegenden Hüft- und Kniegelenkendoprothese

**Abb. 3:**
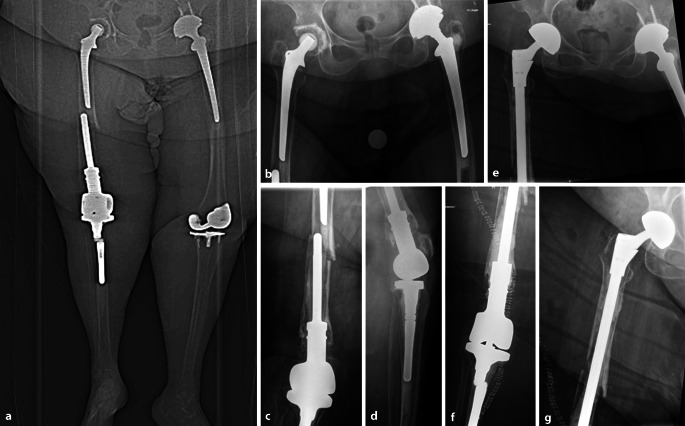
Fallbeispiel: 74 Jahre, weiblich, Body-Mass-Index 47 kg/m^2^. Die Patientin ist im Rollstuhl mobil („low demand“). Der Transfer erfolgte bislang über die linke Seite. Sturzgeschehen beim Aussteigen aus einem Pkw. **a** Topogramm der CT mit Darstellung des eindrücklichen Weichteilmantels. Es zeigt sich eine interprothetische Femurfraktur (Unified-Classification-System-Typ D) bei einliegender, nicht gelockerter, vollzementierter Hüftprothese und zementiertem distalem Femurersatz. Der interprothetische Abstand beträgt lediglich wenige Zentimeter. Weite Konfiguration des Markraumes mit ausgedünnter Femurkortikalis. **b–d** Interprothetische Fraktur mit gelockertem distalen Femurersatz bei festsitzender, zementierter tibialer Komponente. Vorbestehende Patella alta. **e–g** Postoperative Röntgenkontrolle nach Revisionsoperation mit Wechsel auf eine zementfreie tripolare Hüftgelenkpfanne (Fa. Link, Typ BiMobile) sowie Implantation einer Durchsteckprothese mit Erhalt der tibialen Komponente (Fa. Implantcast, Typ MUTARS (Fa. Implantcast, Buxtehude, Deutschland)). Postoperativ wurde die schmerzadaptierte Belastung freigegeben

Liegt eine IFF zwischen einer fest verankerten Hüfttotalendoprothese und einer nicht gelockerten femoral schaftverankerten Kniegelenkprothese vor, so wäre dies als *Typ IIIB* zu klassifizieren. Empfohlen wird von Pires et al. [23] in diesem Fall der totale Femurersatz oder eine langstreckige winkelstabile Plattenosteosynthese mit Knochentransplantat. Die Invasivität eines vollständigen Wechsels zweier fest einliegender femoral verankerter Schäfte ist hier sicherlich in der Risiko-Nutzen-Abwägung zu berücksichtigen. Wenn die Frakturmorphologie und die individuelle Konstitution des Patienten es erlauben, wäre als individueller Therapieansatz auch die Implantation eines sogenannten Rescue-Sleeves eine operative Alternative (Abb. 2). Zu bedenken gilt bei dieser operativen Strategie allerdings, dass es sich um ein individuell angefertigtes Implantat handelt. Die Konfiguration der einliegenden Implantate (z. B. die Dicke der jeweiligen Schaftspitzen) sind im Vorfeld zu eruieren und für die Herstellung des Sleeves essenziell, um dessen Verankerung zu ermöglichen. Der Hersteller benötigt in der Regel mehrere Wochen für die Anfertigung, wodurch diese Therapieoption sicherlich nichts für die akute Fraktursituation ist. Wenngleich die Rescue-Sleeves eine gute Belastbarkeit ermöglichen, so beschreiben Abdelaziz et al. [33] in ihrer Studie ein mechanisches Implantatversagen in 21,7 % über einen Nachuntersuchungszeitraum von durchschnittlich 4 Jahren. In der akuten Fraktursituation bedarf es einer zeitgerechten Versorgung, welche nach Möglichkeit die zügige Mobilisation des Patienten ermöglicht (Abb. 3). Das Risiko von perioperativen Komplikationen ist per se durch das eingebrachte Fremdmaterial erhöht und wird durch die individuellen Risikofaktoren des Patienten entsprechend moduliert.

**Abb. 4:**
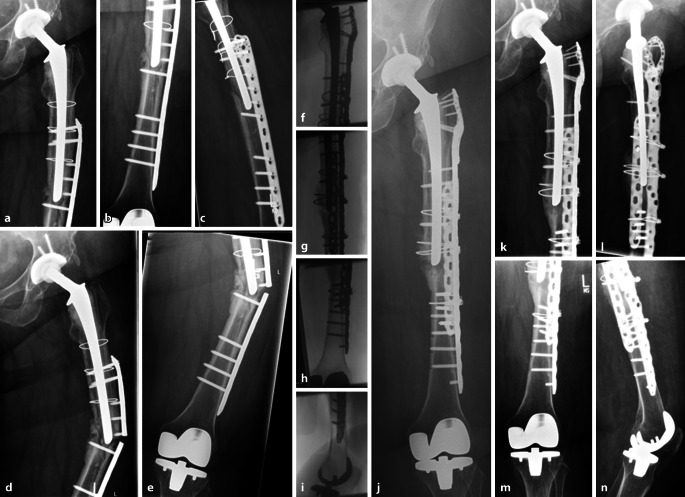
Fallbeispiel: 72 Jahre, weiblich, Body-Mass-Index 42 kg/m^2^. **a–c** Postoperative Röntgenkontrolle nach extern osteosynthetisch versorgter interprothetischer Femurschaftfraktur (Fa. Synthes, LCP distal femur plate (Fa. Synthes, West Chester Pennsylvania, USA)) bei nicht gelockerter teilzementierter Revisionshüfttotalendoprothese sowie nicht gelockertem bikondylärem Oberflächenersatz des Kniegelenkes. **d,** **e** Im Verlauf kommt es nach 8 Monaten – mit zwischenzeitlich mehrmonatiger Entlastung – zur periimplantären Refraktur bei ausbleibender knöcherner Konsolidierung des Femurschaftes. **f–i** Intraoperative Bildwandlerkontrolle nach langstreckiger winkelstabiler Revisionsosteosynthese (Fa. Zimmer, NCB proximal femur plate + trochanter plate) sowie additiver winkelstabiler 90°-Doppelplattenosteosynthese (Fa. Synthes, LCP GF) und Anlagerung von allogener Spongiosa. **j** Postoperative Kontrolle nach Re-Osteosynthese. **k–n** Postoperative Verlaufskontrolle nach 5 Monaten mit Nachweis der konsolidierten interprothetischen Femurschaftfraktur

**Abb. 5:**
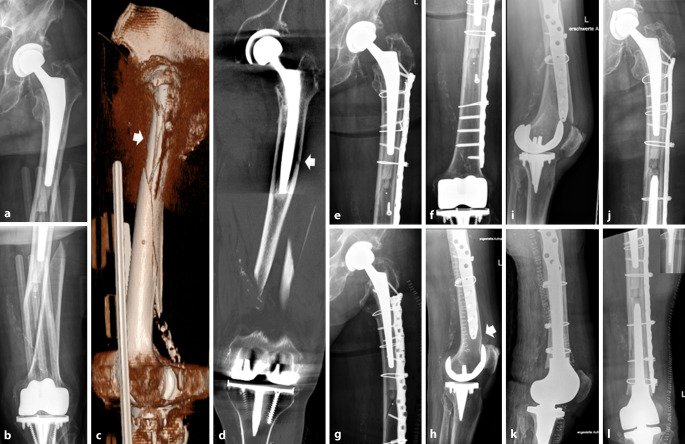
Fallbeispiel: 90 Jahre, männlich. **a,** **b** Konventionell radiologisch zeigt sich eine langstreckige interprothetische Spiralfraktur bei nicht gelockerter teilzementierter Hüfttotalendoprothese und festsitzendem bikondylärem Oberflächenersatz. **c,** **d** In der erweiterten Schnittbildgebung mittels CT zeigt sich eine mehrfragmentäre Frakturmorphologie mit Ausläufern nach proximal bis unterhalb des Trochanter-major-Massivs (*Pfeil*). **e–h** Postoperative Kontrolle nach langstreckiger winkelstabiler Plattenosteosynthese (Fa. Zimmer, NCB proximal femur plate) des Femurschaftes. Ganz distal zeigt sich in der lateralen Projektion ein fissuraler Frakturausläufer der ventralen Femurkortikalis (*Pfeil*). **i** Erneute notfallmäßige Vorstellung 6 Wochen nach der osteosynthetischen Versorgung bei stattgehabtem Sturzereignis aus dem Pflegebett. Konventionell radiologisch zeigt sich eine dorsale Abscherfraktur des distalen Femurs. **j–l** Postoperative Kontrolle nach Revisionsoperation mit Resektion des distalen Femurs und Re-Osteosynthese mit Rekonstruktion der femoralen Schaftkonfiguration mittels Cable-Ready-Cerclagen über die einliegende Plattenosteosynthese sowie Implantation eines modularen zementierten distalen Femurersatzes mit gekoppelter Kniegelenkendoprothese (Fa. Link, Typ Mega C). Der femorale Abstand der Schaftspitzen beträgt weniger als 11 cm, allerdings stabilisiert die langstreckige Plattenosteosynthese protektiv. Postoperativ wurde die schmerzadaptierte Belastung freigegeben

Zeigt sich eine IFF bei fest einliegender Hüftprothese und distal fest einliegendem bikondylärem Oberflächenersatz im Sinne einer Fraktur *Typ IA*, so kann diese durch eine osteosynthetische Versorgung adressiert werden (Abb. 4). Die Auswahl des Osteosynthesematerials ist hierbei entscheidend. Walcher et al. [34] analysierten anhand von 38 Sawbone-Femora (4. Generation), welche intramedullär bis 23 mm aufgereamed wurden, um ein altersentsprechendes Kollektiv zu generieren, die Plattenpositionierung bei der Versorgung von IFF. Im Bereich des proximalen Femurs wurde ein zementierter Schaft implantiert. Additiv wurde eine laterale Osteosynthese des Femurs vorgenommen (Fa. Zimmer, NCB distal femur plate (Fa. Zimmer, NCB distal Femur plate, Warsaw Indiana, USA)). In 2‑cm-Schritten wurde die Plattenosteosynthese mit einem Abstand zum proximal einliegenden Schaft von 8 cm unterhalb bis 6 cm den Schaft überlappend positioniert. Es folgte eine zyklische Belastung und Torsion des Präparates bis hin zur maximalen „load to failure“. Die Autoren beobachteten ein frühzeitiges Versagen mit Fraktur des Präparates bei einem Abstand bzw. einer Überlappung zwischen Platte und Schaftspitze von nur 2 cm. Walcher et al. [34] schlussfolgerten anhand ihrer Analyse, dass eine Überlappung bzw. ein Abstand zwischen den Implantaten von mindestens 6 cm einzuhalten ist, um das Frakturrisiko zu reduzieren. Bei Querfrakturen unterhalb der Schaftspitze der einliegenden Hüfttotalendoprothese bzw. oberhalb bei femoral schaftverankerter Kniegelenkprothese ist eine Doppelplattenosteosynthese zum Schutz der lateralen Platte sowie zur Erhöhung der Osteosynthesestabilität indiziert. Auch die Anlagerung von autologem oder allogenem Knochen ist optional zu erwägen [35]. Wenngleich die Osteosynthese bei einer Fraktur Typ IA die mutmaßlich richtige operative Versorgungsstrategie darstellt, so können, wie eingangs erwähnt, individuelle Faktoren den weiteren Behandlungsverlauf beeinflussen. Insbesondere sollte bei jeglicher Versorgung von IFF die anzunehmende Compliance des Patienten bedacht werden. Eine Teilbelastung des mitunter betagten Patientenkollektivs ist oftmals nur schwer zu realisieren. Somit ist auch das Versagen der Osteosynthese ein nicht gänzlich unwahrscheinliches Szenario. In diesen Fällen müssen individuelle Lösungen gefunden werden, um die Wiederherstellung der Mobilität des Patienten zu erreichen (Abb. 5).

## Wie sind das Ergebnis und die Mortalität nach operativer Versorgung von IFF?

Neitzke et al. [35] konnten in ihrer retrospektiven Analyse über einen Zeitraum von 2008 bis 2021 insgesamt 76 IFF (Ø 75 Jahre; 78 % Frauen; BMI 30 kg/m^2^) einschließen. In 71 % der Fälle erfolgte – über den 2‑jährigen Nachbeobachtungszeitraum – keine erneute operative Revision. Dieses Ergebnis ist vergleichbar mit den Ergebnissen der Arbeitsgruppe um Tibbo [36], welche in ihrer Studienkohorte über einen 2‑jährigen Nachbeobachtungszeitraum in 81 % der Fälle keine operative Revision beobachteten. Die häufigsten Gründe für eine Revision in der Studie von Neitzke et al. [35] waren die Infektion, die Refraktur sowie die ausbleibende knöcherne Heilung. Bei einer IFF mit femoral schaftverankerter Kniegelenkprothese zeigte sich ein höheres operatives Revisionsrisiko. Ein signifikanter Unterschied zwischen Osteosynthese und Revisionsprothese ließ sich anhand des Kollektivs nicht ableiten. Die vergleichsweise hohe Rate an Majorkomplikationen (24 %) in der Arbeit von Neitzke et al. zeigt sich ebenfalls in anderen Studien [8]. Hingegen gaben Miettinen et al. [8] in ihrer Arbeit (31 IFF; Ø 77,1 Jahre; 74 % Frauen) als häufigsten Revisionsgrund die ausbleibende knöcherne Heilung an.

Neben mechanischen Faktoren (z. B. Wahl und Positionierung der Osteosynthese) müssen zudem auch individuelle biologische Einflussfaktoren, welche eine knöcherne Konsolidierung potenziell kompromittieren, bedacht werden [37]. Unabhängig von der chirurgischen Technik im Rahmen der operativen Frakturversorgung können der Grad der Frakturdislokation, Voroperationen oder auch der traumatisch bedingte Weichteilschaden die Knochenheilung negativ beeinflussen. Des Weiteren ist auch der alternde Knochen, für welchen Alimy et al. [38] eine zunehmende Mineralisation der Osteozytenlakunen aufzeigen konnten, aus Sicht der Knochenqualität ein möglicher Risikofaktor für die eingeschränkte Konsolidierung der Fraktur. Selbstverständlich sollte auch im Rahmen der operativen Frakturversorgung eine sorgsame Präparation und Frakturreposition – mit so geringem iatrogenen Weichteiltrauma wie möglich – sichergestellt werden. Ungeachtet vom Revisionsgrund zeigt die hohe Komplikationsrate einmal mehr die Komplexität und Schwierigkeit der IFF auf.

Ein weiterer Ergebnisparameter nach operativer Versorgung von IFF ist die Mobilität des Patienten im postoperativen Verlauf. Die Wiedererlangung der Gehfähigkeit nach operativ versorgter IFF wird in der Literatur mit einer Spanne von 56–94 % beziffert [28, 35, 39], welches sicherlich auch die Problematik des geriatrischen Patientenkollektivs reflektiert.

Unter Berücksichtigung der vergleichsweise hohen Komplikationsrate und der im Mittel eher reduzierten wiedererlangten eigenständigen Mobilität erscheint es plausibel, dass die Mortalität der eher betagten Patienten entsprechend hoch ist. Miettinen et al. [8] beschrieben in ihrer Arbeit mit insgesamt 31 IFF (Ø 77,1 Jahre; 74 % Frauen), dass bis zum Ende des Nachuntersuchungszeitraumes 65 % der Patienten verstorben waren (1-Jahres-Mortalität 3,2 %; 10-Jahres-Mortalität 50,4 %). Die durchschnittliche Zeit bis zum Tod nach IFF betrug 5,7 Jahre [7]. Füchtmeier et al. [40] analysierten insgesamt 50 Patienten (Ø 5,7 Jahre Nachuntersuchungszeitraum) nach operativ versorgter IFF. Die 1‑Jahres-Mortalität betrug 14 %, wobei Frakturmorphologie und Versorgungstrategie keinen signifikanten Effekt zeigten. Dies ist vergleichbar mit den Ergebnissen von Marr et al. [41], die die 1‑Jahres-Mortalität in ihrer retrospektiven Analyse von 70 IFF mit 10 % bezifferten, wobei das Risiko, nach osteosynthetischer Versorgung zu versterben, in dieser Studienkohorte erhöht war.

## Fazit für die Praxis

- Die „load to failure“ reduziert sich durch die veränderten biomechanischen Eigenschaften des Femurs nach Schaftimplantation.
- Bei proximal und distal einliegendem intramedullärem Implantat steigt der „Stress“ interimplantär und konsekutiv ist das Frakturrisiko erhöht.
- Das Frakturrisiko wird eher durch die suffiziente Implantatverankerung und die kortikale Stärke (Dicke/Fläche) determiniert.
- Ein interprothetischer Abstand von ≥ 11 cm scheint das Risiko für eine Fraktur signifikant zu reduzieren.
- In den Entscheidungsprozess bei der Frakturversorgung müssen neben der verbliebenen Implantatstabilität auch die Knochenqualität und etwaige Komorbiditäten mit einbezogen werden.
- Die Expertise der Behandelnden in verschiedenen Osteosyntheseverfahren sowie in der Revisionsendoprothetik scheint unabdingbar.

## Abkürzungen

BMD: „Bone mineral density“
BMI: Body-Mass-Index
EPRD: Endoprothesenregister Deutschland
IFF: Interprothetische Femurfraktur
ORIF: „Open reduction and internal fixation“
PFF: Periprothetische Femurfraktur
UCS: Unified Classification System
